# Fine-scale geographic difference of the endangered Big-headed Turtle (*Platysternon megacephalum*) fecal microbiota, and comparison with the syntopic Beale’s Eyed Turtle (*Sacalia bealei*)

**DOI:** 10.1186/s12866-024-03227-2

**Published:** 2024-02-29

**Authors:** Jonathan J. Fong, Yik-Hei Sung, Li Ding

**Affiliations:** 1 Science Unit, Lingnan University, Hong Kong, China; 2 School of Allied Health Sciences, University of Suffolk, 19 Neptune Quay, Ipswich, IP4 1QJ UK; 3 Ministry of Education Key Laboratory for Ecology of Tropical Islands, College of Life Sciences, Hainan Normal University, Haikou, China

**Keywords:** Gut microbiome, Common core microbiome, Turtle conservation, *Citrobacter*

## Abstract

**Background:**

Studies have elucidated the importance of gut microbiota for an organism, but we are still learning about the important influencing factors. Several factors have been identified in helping shape the microbiome of a host, and in this study we focus on two factors—geography and host. We characterize the fecal microbiota of the Big-headed Turtle (*Platysternon megacephalum*) and compare across a relatively fine geographic scale (three populations within an 8-km radius) and between two syntopic hosts (*P. megacephalum* and *Sacalia bealei*). Both species are endangered, which limits the number of samples we include in the study. Despite this limitation, these data serve as baseline data for healthy, wild fecal microbiotas of two endangered turtle species to aid in conservation management.

**Results:**

For geography, the beta diversity of fecal microbiota differed between the most distant sites. The genus *Citrobacter* significantly differs between sites, which may indicate a difference in food availability, environmental microbiota, or both. Also, we identify the common core microbiome for *Platysternon* across Hong Kong as the shared taxa across the three sites. Additionally, beta diversity differs between host species. Since the two species are from the same site and encounter the same environmental microbiota, we infer that there is a host effect on the fecal microbiota, such as diet or the recruitment of host-adapted bacteria. Lastly, functional analyses found metabolism pathways (KEGG level 1) to be the most common, and pathways (KEGG level 3) to be statistically significant between sites, but statistically indistinguishable between species at the same site.

**Conclusions:**

We find that fecal microbiota can significantly differ at a fine geographic scale and between syntopic hosts. Also, the function of fecal microbiota seems to be strongly affected by geographic site, rather than species. This study characterizes the identity and function of the fecal microbiota of two endangered turtle species, from what is likely their last remaining wild populations. These data of healthy, wild fecal microbiota will serve as a baseline for comparison and contribute to the conservation of these two endangered species.

**Supplementary Information:**

The online version contains supplementary material available at 10.1186/s12866-024-03227-2.

## Introduction

The gut microbiota plays essential roles for the host in terms of metabolism, immunity, growth, and development [1–3]. In gut microbiota studies, a major goal is to identify the factors that shape the gut microbiota and their relative importance. Some factors typically identified are host diet [4–6], gut anatomy [7], host phylogeny [8–10], reproductive stage [11], season [12, 13], altitude [14], and geography [15, 16]. A study across vertebrates found that the most important factor varied for different vertebrate lineages, with a strong correlation between microbial community, host diet, and host phylogenetic distance in non-flying mammals and weak correlation in flying mammals [17].

In this study, we evaluate the effect of two factors on the gut microbiota: geography and host. A recent study of fish species along the Yellow River in China found these two factors to be important in shaping the gut microbiota [18]. For geography, previous studies found significant influence at the level of continent [15, 19], country [11, 20], and finer scale [16, 21]. In our study, we characterize the fecal microbiota of the Big-headed Turtle (*Platysternon megacephalum*) and see whether the microbiota varies across a relatively fine geographic scale (three populations within an 8-km radius).

Next, we investigate the influence of a host on its gut microbiota. Some studies have found that gut microbiota varies according to the host phylogeny [8–10], indicating the importance of evolutionary forces and the development of a host-microbiota symbiotic relationship. Other studies have found that gut microbiota can be similar for different host species due to similar diet [17, 22, 23] or ecology [17, 24], indicating the importance of the environmental factors. In our study, we evaluate the impact of a host by comparing the fecal microbiota of two species: *P. megacephalum* and Beale’s Eyed Turtle (*Sacalia bealei*). To minimize the effect of the environment, we choose a site where the two species are syntopic.

Studies of the two target species are importance because they are endangered. In fact, turtles in Asia are being pushed towards extinction due to unsustainable hunting for the food and pet trades [25]. *Platysternon megacephalum* and *S. bealei* are listed as critically endangered and endangered on the IUCN Red List, respectively [26], and wild populations are rare. However, wild populations persist in Hong Kong in part because they are protected by legislation (Cap. 170). This provides a unique opportunity to study the fecal microbiota of what is likely the last remaining wild populations of these two species. These findings contribute to our understanding of how geography and host influence the gut microbiota. Additionally, we gather baseline data on the identity and function of healthy, wild fecal microbiotas, which will help in the conservation and management these two endangered turtle species.

## Methods

### Sampling

All methods used in this study were approved and performed in accordance with the relevant guidelines and regulations of Lingnan University Research Committee (Sub-Committee on Research Ethics and Safety), and permission to capture, handle, and take samples from these endangered species was approved by the Agriculture, Fisheries and Conservation Department of the Hong Kong Special Administrative Region Government, China (Permit # (94) in AF GR CON 09/50 pt. 29). A total of 16 wild *P. megacephalum* were collected from three sites (Site X: 4 samples, Site Y: 7 samples, Site Z: 5 samples) in Hong Kong using aquatic traps baited with dead fish [27]. A study on humans found that a large shift in diet (vegetarian to carnivore diet) can lead to a quick change in gut microbiota (3–4 days) [5]; we do not know if or how the bait in traps affect the results of our study, but alternative hunting methods without bait (e.g., active searching) are inefficient for capture. So, we are careful to consider the potential effect of bait in our interpretation of the results. We do not provide detailed locality data due to the endangered status of these turtle species. We use these samples to characterize the fecal microbiota of *P. megacephalum*, as well as investigate geographic differences in fecal microbiota. Sites Y and Z are approximately 1 km straight line distance from each other and are located in different drainages of the same mountain. From ongoing mark-recapture work, individuals are known to move between these two sites. Site X is ~ 8 km from the other sites and is found on a different mountain.

We include three samples of wild *S. bealei* sequenced in a previous study (WS1.1, WS1.2, WS1.3) [6]. These *S. bealei* are from Site X and we compare with *P. megacephalum* from the same site to investigate host differences in fecal microbiota. Since we are dealing with an endangered species with small wild population sizes, the sample sizes are relatively low compared to other microbiota studies. However, we have shown in a previous study [6] that such sample sizes are sufficient to elucidate important trends.

We collected fecal samples following Fong et al. [6]. Briefly, individuals were placed into containers with a wire mesh floor, where the excreted feces would fall through the wire mesh floor, preventing the individual from stepping on and contaminating the sample. Feces for each individual were collected within 24 h of capture and immediately placed in a sterile 2 mL tube and frozen at -80 °C. After sample collection, the container was sterilized with a 10% bleach solution.

### DNA extraction and PCR amplification

Total DNA of fecal samples was extracted using a E.Z.N.A.® Soil DNA Kit (Omega Bio-Tek; Norcross, Georgia, USA). The concentration and purification of DNA were measured using a NanoDrop 2000 (Thermo Scientific; Wilmington, USA). The V3-V4 hypervariable region of the bacterial 16S rRNA gene was amplified with primers 338F (5’- ACTCCTACGGGAGGCAGCAG-3’) and 806R (5’-GGACTACHVGGGTWTCTAAT-3’). PCR reactions for each sample were performed in triplicate in 20 µL reactions containing 4 µL of 5 × FastPfu Buffer, 2 µL of 2.5 mM dNTPs, 0.8 µL of each primer (5 µM), 0.4 µL of FastPfu polymerase, and 10 ng of template DNA. The following thermal cycler program was used for amplification: 3 min at 95 ºC; 27 cycles of 30 s at 95 ºC, 30 s at 55 ºC, and 45 s at 72 ºC; and a final extension at 72 ºC for 10 min. The PCR products were purified using the AxyPrep DNA Gel Extraction Kit (Axygen Biosciences; Union City, California, USA) and quantified using a QuantiFluor ™-ST (Promega; Madison, Wisconsin, USA).

### High-throughput sequencing and data processing

Purified amplicons were pooled in equimolar concentrations (11 ng DNA for each sample) and paired-end sequenced (2 × 300) on an Illumina MiSeq platform (Illumina; San Diego, California, USA) according to the standard protocols of Majorbio Bio-pharm Co., Ltd. (Shanghai, China). The raw reads of new samples were submitted to the NCBI Sequence Read Archive (SRA) database (accession number: PRJNA824218). Data for *S. bealei* were previously submitted to the SRA database (accession number: PRJNA623155).

Raw FASTQ file reads were quality-filtered with Trimmomatic [28] by truncating reads at any site receiving an average quality score < 20 over a 50 bp sliding window, and removing reads if they contained ambiguous bases or primer sites had > 2 nucleotide mismatches. Reads were then merged with FLASH [29] if they had matching overlap longer than 10 bp. All samples were rarefied to the sample with the lowest number of reads. Operational taxonomic units (OTUs) were clustered with a threshold of 97% similarity cutoff using UPARSE v.7.1 [30] and chimeric sequences were identified and removed using UCHIME [31]. Bacterial taxonomy was assigned to the species level using the SILVA database (Release 138.1; http://www.arb-silva.de), removing non-relevant OTUs (eukaryote, mitochondria, chloroplast).

### Alpha and beta diversity analyses

We perform all analyses separately for two datasets—*P. megacephalum* from the three sites (*Platysternon* dataset), and *P. megacephalum* and *S. bealei* from Site X (*Platysternon*/*Sacalia* dataset). To determine whether sequencing depth was sufficient to cover the expected number of OTUs at the level of 97% sequence similarity, rarefaction curves were created in Mothur v.1.30.1 [32].

Four alpha diversity indices (ACE, Chao1, Shannon, Simpson) were calculated in Mothur [32]. The normality of datasets was tested using a Kolmogorov–Smirnov (K-S) test and homogeneity tested using a Homogeneity Variance (H-V) test, using IBM SPSS Statistics 22. If the data are normally distributed and homogenous (both values > 0.05), one-way ANOVA was used to evaluate whether alpha diversity indices are statistically significant of the *Platysternon* dataset (three groups), and Student’s t-test for the *Platysternon*/*Sacalia* dataset (two groups). Otherwise, a Kruskal–Wallis H test or Wilcoxon rank-sum test was used, respectively.

Data were visualized by principal coordinate analysis (PCoA) based on weighted or unweighted UniFrac distances. Analysis of Similarities (ANOSIM) was performed to determine the differences among sites [33] as a metric of similarity between the bacterial communities based on the abundance of OTUs between samples. We used R [34] to produce PCoA and Venn diagrams, as well as run ANOSIM.

### Bacteria composition and relative abundance

Community structure was analyzed at three taxonomic levels (phylum, family, and genus). For the *Platysternon* dataset, first, significant difference between the three sites was evaluated using either a one-way ANOVA or Kruskal–Wallis H test depending on the distribution of the data. Second, due to the similarity between Site Y and Z, we combine data from these two sites and test again for significant differences (Site X vs. Sites Y/Z) using either a Student’s t-test or Wilcoxon rank-sum test. For the *Platysternon*/*Sacalia* dataset, significant difference was evaluated using either a Student’s t-test or Wilcoxon rank-sum test. A *P* value < 0.05 was considered to be statistically significant for all analyses.

To identify the taxa that explain the differences within each dataset, we ran a linear discriminant analysis effect size (LEfSe) using the LEfSe software, with the filter value of the LDA score set as 2 or 4 [35].

### Functional analysis

We predicted functional profiles of the 16S rRNA datasets. We base these predictions on the SILVA database by first converting the taxonomic lineages of prokaryotes in the KEGG database using Tax4Fun. Then we performed KEGG functional annotation of the 16S rRNA gene sequences.

## Results

### Analysis of rRNA sequencing results

The general information of the samples and sequencing is in Table S1. The number of quality-filtered sequences obtained for each *P. megacephalum* sample is 37,359–59,211, for a total of 789,806 sequences (203,613 reads from Site X; 317,403 reads from Site Y; and 228,202 reads from Site Z). Rarefaction curves reached the saturation phase (Figure S1), indicating that there is sufficient sampling depth.

### *Platysternon* dataset

The four alpha diversity indices (Shannon, Simpson, ACE, Chao1) are displayed in Table 1 and Figure S2. The data are normally distributed and homogenous (all K-S and H-V tests *P* > 0.05), so one-way ANOVA was used for tests of statistical significance. Site X had higher values for Simpson, ACE, and Chao1, while Sites Y and Z had a higher value for Shannon. However, none of these differences are significant (*P* > 0.05).

**Table 1:** Comparison of alpha diversity indices of fecal microbiota between the Big-Headed Turtle (*Platysternon megacephalum*) from three different sites and *P. megacephalum* and *Sacalia bealei* from the same site (Site X). Data are mean ± SD. All data are homogenous and normally distributed, so one-way ANOVA or Student’s t-test was used to test for significance. There are no significant differences in indices between *Platysternon* sites, and between *Platysternon* and *Sacalia*

Index	Site X	Site Y	Site Z	*P value*			
				Site X vs Site Y	Site X vs Site Z	Site Y vs Site Z	*Platysternon* vs *Sacalia*
Shannon	2.26 ± 0.38	2.50 ± 0.74	2.55 ± 0.87	0.562	0.558	0.920	0.094
Simpson	0.22 ± 0.08	0.20 ± 0.12	0.17 ± 0.14	0.726	0.525	0.712	0.081
ACE	218.49 ± 102.26	155.26 ± 74.40	213.68 ± 84.78	0.258	0.941	0.228	0.463
Chao1	218.35 ± 107.88	151.82 ± 71.30	199.12 ± 91.35	0.245	0.780	0.336	0.499

**Fig. 1:**
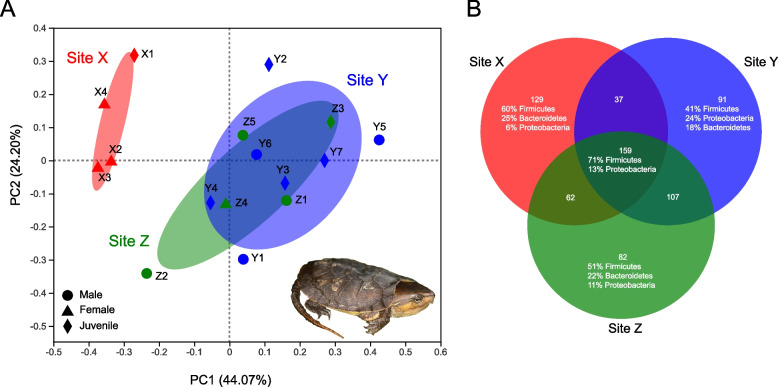
Beta diversity and Analysis of Similarities (ANOSIM) analyses. **A** Principal coordinates analysis (PCoA) plot of beta diversity based on weighted UniFrac distances for bacterial communities at the OTU level. The main coordinates (PC1 and PC2) are represented in the axes, and their relative contributions are denoted by the percentage in parentheses. **B** Venn diagram showing the unique and shared OTUs

Beta diversity analyses are illustrated in the PCoA plot (Fig. 1A). A total of 68.27% of the variance is explained by PC1 and PC2. Samples clustered based on site, instead of sex and age class. Individuals from Sites Y and Z overlap in the ordination plot, while individuals from Site X are distinct from Y and Z, indicating that the bacterial communities of Site X are different from Sites Y and Z. ANOSIM results support this conclusion, with significant differences found between Site X and Sites Y and Z: Sites X and Y (R = 0.77, *P* = 0.004); Sites X and Z (R = 0.68, *P* = 0.01); Sites Y and Z (R = -0.15, *P* = 0.93); Sites X, Y, and Z (R = 0.35, *P* = 0.004). The shared and unique microbiota between sites are displayed using a Venn diagram (Fig. 1B).

**Fig. 2:**
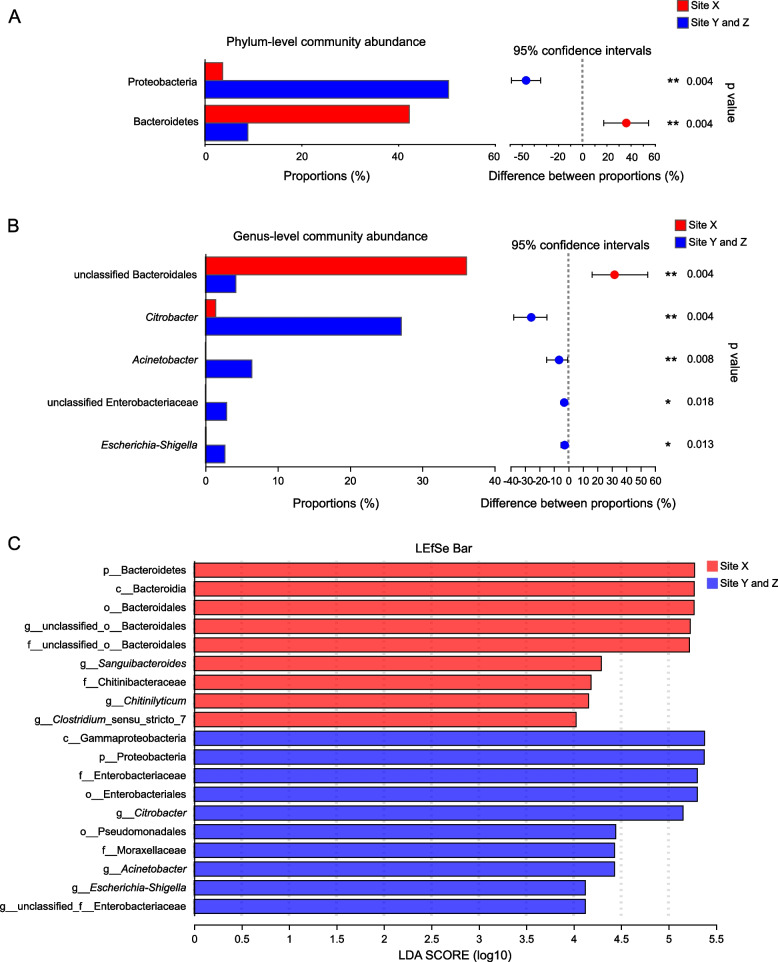
Comparison of bacterial community between Site X and Sites Y/Z. **A** Phylum-level community abundance bar plots. **B** Genus-level community abundance bar plots. The taxa significantly different between sites are indicated (*, *P* < 0.05; **,* P* < 0.01). *P* values are based on Student’s t-test or Wilcoxon rank-sum test, depending on the distribution of the data. **C** Linear discriminant analysis effect size (LEfse). The bar graph of LDA scores showing the taxa statistically different between Sites X, Y, and Z. The degree of influence of a taxon is expressed by the length of the bar. Only taxa meeting an LDA significant threshold > 4 are shown

The 667 OTUs are classified into 21 phyla, 30 classes, 62 orders, 108 families, and 244 genera. For the analyses of community abundance, the results from the two datasets (comparison three individual sites, comparison of Site X vs. Sites Y/Z) were largely concordant, but we focus on the comparison of Site X vs. Sites Y/Z because the patterns are more clear and statistically well-supported (Fig. 2). The results from the analyses comparing the three individual sites can be found in the supplementary materials (phylum level [Figure S3], family level [Figure S4], genus level [Figure S5], LEfSE [Figure S6]). For the relative abundance at the phylum level, Site X has significantly more Bacteroidetes, while Sites Y/Z have significantly more Proteobacteria (Fig. 2A).

For the relative abundance at the genus level, Site X has significantly more unclassified Bacteroidales (Fig. 2B). We use BLAST to refine the identification of the three most abundant unclassified Bacteroidales OTUs (OTU431, OTU169, OTU436), and found high similarity with the genera *Macellibacteroides*, *Paludibacter*, and Rikenellaceae. In contrast, the genera significantly higher in Sites Y/Z compared to Site X are *Citrobacter*, *Acinetobacter*, unclassified Enterobacteriaceae, and *Escherichia-Shigella* (Fig. 2B).

From the LEfSe analysis, nine nested taxa from Site X and 10 from Sites Y/Z are identified to explain the differences between the two groups (Fig. 2C). Generally, as seen in our other analyses, Site X is characterized by taxa in phylum Bacteroidetes, while Sites Y/Z by taxa in the phylum Proteobacteria (Fig. 2C).

### *Platysternon*/*Sacalia* dataset

The four alpha diversity indices (Shannon, Simpson, ACE, Chao1) are displayed in Figure S7. All K-S and H-V tests are not significant (*P* > 0.05), indicating that these data are normally distributed and homogenous, so Student’s t-test was used for tests of statistical significance. *Platysternon* has a higher value for Simpson, while *Sacalia* has a higher value for Shannon, ACE, and Chao1. However, none of these differences are significant (*P* > 0.05).

**Fig. 3:**
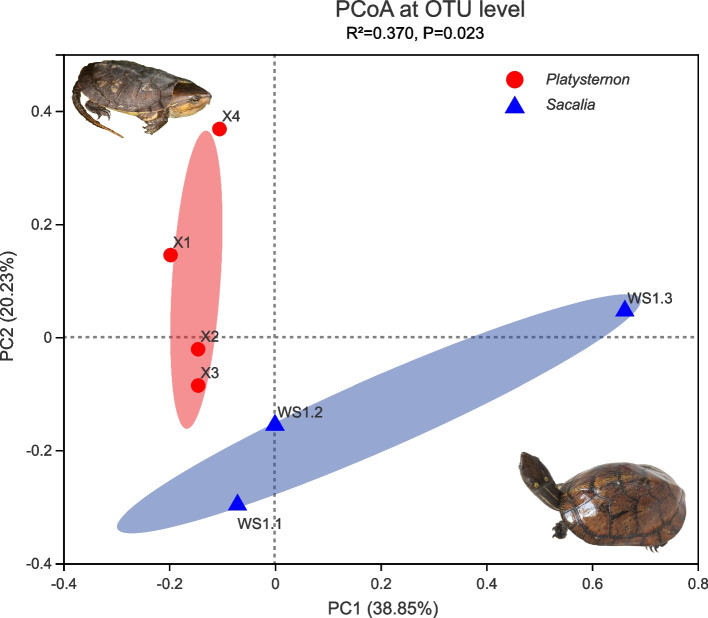
Principal coordinates analysis (PCoA) plot of beta diversity for *Platysternon* and *Sacalia*. Plot is based on weighted UniFrac distances for bacterial communities at the OTU level. The main coordinates (PC1 and PC2) are represented in the axes, and their relative contributions are denoted by the percentage in parentheses

**Fig. 4:**
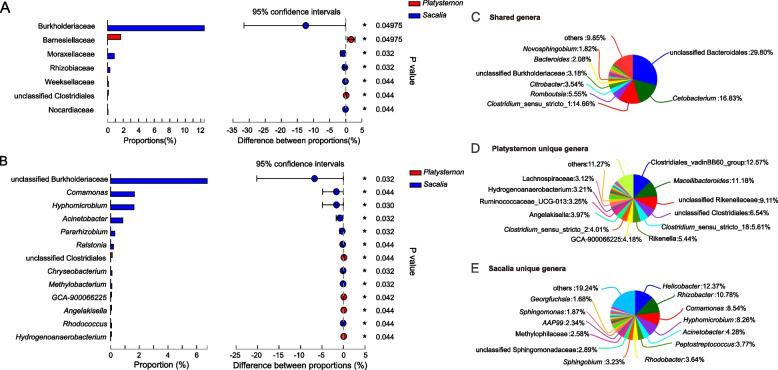
Comparison of bacterial community between *Platysternon* and *Sacalia*. The significant difference between *Platysternon* and *Sacalia* at the level of (**A**) family and (**B**) genus. **C** Shared genera between *Platysternon* and *Sacalia.* (**D**) Unique genera to *Platysternon*, (**E**) Unique genera to *Sacalia*

Beta diversity analyses are illustrated in the PcoA plot (Fig. 3). A total of 59.08% of the variance is explained by PC1 and PC2. There is no overlap between *Platysternon* and *Sacalia* from the same site, supported by the ANOSIM analysis (R = 0.463, *P* = 0.023). For taxa significantly different between *Platysternon* and *Sacalia*, we identify zero phyla, seven families (Fig. 4A), and 13 genera (Fig. 4B). Shared (Fig. 4C) and unique genera for *Platysternon* (Fig. 4D) and *Sacalia* (Fig. 4E) are also identified. The top three shared genera between the two species are unclassified Bacteroidales (29.80% of the total shared genera), *Cetobacterium* (16.83%) and *Clostridium* (14.66%). The top three genera unique to *Platysternon* are Clostridiales, *Macellibacteroides* and unclassified Rikenellaceae, while for *Sacalia* are *Helicobacter*, *Rhizobacter*, and *Comamonas*.

### Functional analysis

**Fig. 5:**
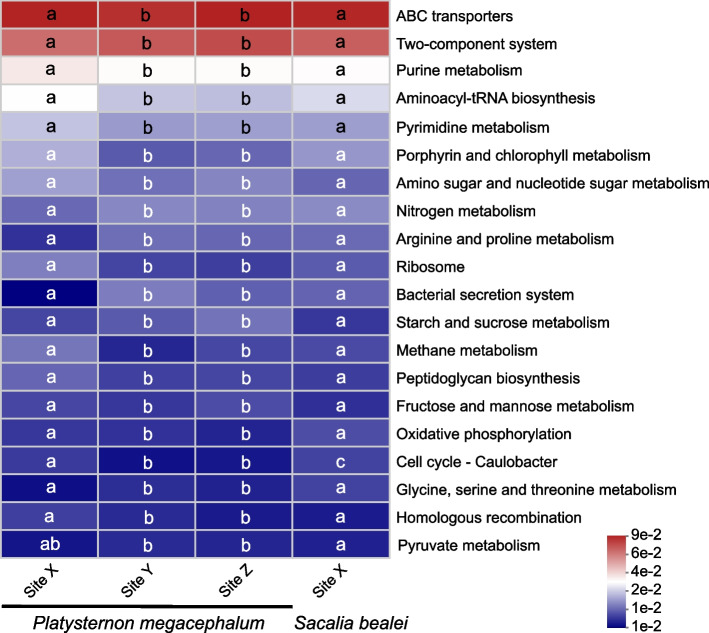
The functional analysis of the gut microbiota. Heat map showing the top 20 predicted pathways of KEGG function abundance. The columns represent groups, and the rows represent the function. The relative change of different functions is represented by the color gradient across rows, and different lowercase letters indicate significant differences between groups

The predicted functions of the bacterial community in *Platysternon*/*Sacalia* were analyzed by KEGG pathways. According to KEGG level 1 pathway, many predicted functions were associated with five main categories; pathways regarding metabolism were the most common and at similar proportion for both *Platysternon* (58.71–60.06%) and *Sacalia* (60.23%) (Figure S8). The level 3 KEGG pathway database showed that the pathways are diverse, with the two most common being for ABC transporters and two-component system (Fig. 5). When comparing the relative abundance of pathways between groups, Site Y and Z were statistically indistinguishable, as well as both species at Site X. The only two exceptions are “Cell cycle-Caulobacter” and “Pyruvate metabolism” (Fig. 5).

## Discussion

In this study, we characterize of the wild fecal microbiota of the endangered Big-headed Turtle (*P. megacephalum*) and identify significant geographic difference in fecal microbiota at a relatively fine scale (~ 8 km). Additionally, we identify differences in the fecal microbiota of two syntopic species (*Platysternon* and *Sacalia*). We discuss potential causes and effects of such differences, highlight key taxa differing between sites, and discuss the implications of our findings on the study of turtle gut microbiota and conservation.

### Geographic difference in fecal microbiota

A majority of studies investigating the influence of geography on the gut microbiota sample across a broad geographic range across countries or continents [11, 15, 18–20]. We found that the fecal microbiota of *P. megacephalum* differed at a fine-scale geography (three sites within 8 km) (Fig. 1). This finding is similar to Yuan et al. [16], who found the fecal microbiota of tortoises to vary significantly across four sites within ~ 2 km. Geographic differences are typically explained to be a result of different diets, as geography and food availability are related [36]. Yuan et al. [16] proposed a similar explanation to the differences they found; since the four sites were in different fire management units (in a biological station), the differences were likely due to differences in food availability and/or environmental microbiota. It should be noted that our use of bait for capture of individuals could have affected results, but we predict the influence to be small for two reasons. First, we used the same bait at each site (dead fish [27]). If this has any effect, we expect the microbiota to become homogenized and more similar across sites. However, we still found microbiome differences across some sites. Second, feces were collected within 24 h of trapping, which should minimize the impact of ingesting bait; one study on humans found that microbiota change can be quick, but typically occurring after 3–4 days [5].

The similarity between the fecal microbiota between Sites Y and Z make sense when considering the ecology of *P. megacephalum*. Based on a radio-telemetry based study, the 100% minimum convex polygon size for this species is 996 m^2^ [37], which is similar to the distance between Sites Y and Z. Additionally, since these sites are part of a long-term monitoring program, we have observed migration of marked individuals between these two sites; for example, an individual captured from Site Y was later found at Site Z, and vice versa (Sung, unpublished data).

Could diet contribute to the difference in fecal microbiota between Site X and Sites Y/Z? A diet study of *P. megacephalum* using visual fecal analysis found its diet to be dominated by fruit, crabs, mollusks, and insects, and to differ across five sites across Hong Kong [38]. The diets at Sites Y and Z (MS and SH in [38], respectively) were found to be statistically different, while Site X was not included in their study. In contrast, our results infer that the diets of Sites Y and Z are similar, and this difference could be due to the resolution and temporal scale of different diet tracing methods, with visual analysis at the scale of hours to days [39] and gut microbiota longer. Although we do not have systematically collected data on food availability at the different sites, we have generally observed that Site X has lower availability of mollusks and fruit (but higher diversity of fruit) compared to Sites Y and Z. These differences are likely due to differences in habitat—Site X (flatter stream, finer substrate, low canopy cover, high plant diversity) and Sites Y and Z (steeper streams, larger substrate, high canopy cover, low plant diversity). The difference in fecal microbiota we observe in *P. megacephalum* between sites could be a result of food availability, environmental microbiota, or both, and additional field and laboratory studies are needed to determine the cause.

The concept of a core gut microbiome generally refers to the constant portions across individuals, and has five complementary definitions [40]: (1) common core—shared taxa, (2) temporal core—taxa temporally stable across hosts, (3) ecological core—keystone taxa shaping microbial environment, (4) functional core—microbial genes important to function of host, and (5) host-adapted core—taxa with highly conserved association with host. We identify the common core microbiome for *Platysternon* across Hong Kong as the shared taxa across the three sites (Fig. 1B). The shared occupancy of these taxa across individuals and geographic locations indicate that they may be well adapted to *Platysternon* and possess functions that enable their prevalence. However, our analysis cannot determine whether microbial taxa are essential for the host function, as diet and other biotic and abiotic factors may be involved [40]. Our identification of the shared microbial taxa is the first step in identifying the core microbiome of *Platysternon*, which can serve as a hypothesis to test in other gut microbiome studies of *Platysternon* and other freshwater turtles in Asia.

We used three statistical analyses (Kruskal–Wallis H test, one-way ANOVA, and LEfse) to identify OTUs at three taxonomic levels (phylum, family, genus) significantly different between sites. At the phylum level, Bacteroidetes is significantly higher at Site X, while Proteobacteria is significantly higher at Sites Y/Z while (Fig. 2A). Methods to inferring function based on microbiota are developing, but some information can be gleaned from published studies. Bacteroidetes assists in degrading complex macromolecular matter of both plants and animals [3], but is unclear what a difference in Bacteroidetes indicates. Qu et al. [41] studied an invasive and native turtle species to China and found both to have a gut microbiota dominated by Bacteroidetes. In human studies, Bacteroidetes is a dominant component of the gut microbiota, and a high Firmicutes:Bacteroidetes ratio indicating predisposition to disease and obesity [42]. For Proteobacteria, organisms with animal-based diets tend to have a higher proportion of bile-tolerant Proteobacteria [5]. Additionally, Proteobacteria are associated with the breakdown of complex sugars and synthesis of vitamins [3]. This infers that turtles at Sites Y/Z are more carnivorous than turtles at Site X.

At the genus level, unclassified Bacteroideales is significantly higher at Site X, while *Citrobacter*, *Acinetobacter*, unclassified Enterobacteriaceae, and *Esherichia-Schigella* are significantly higher at Sites Y/Z. Based on our BLAST analysis, we refine the unclassified Bacteroidales, to the genera *Macellibacteroides*, *Paludibacter*, and Rikenellaceae. In terms of function, *Macellibacteroides* can decompose cellulose- and hemicellulose-derived sugars [43], but we were unable to find any information on potential function of *Paludibacter* and Rikenellaceae.

For three of the genera more prevalent at Sites Y/Z (*Acinetobacter*, unclassified Enterobacteriaceae, and *Esherichia-Schigella*), they have been linked to stress [44] and disease [45, 46]. For *Citrobacter*, the fourth genus more prevalent at Sites Y/Z, the variation at the phylum-level Proteobacteria among groups is largely driven by differences in the genus *Citrobacter* (Fig. 2B). *Citrobacter* was found to be dominant in the gut of several freshwater and euryhaline fish species: Malaysian Mahseer (*Tor tambroides*; [47]), Nile Tilapia (*Oreochromis niloticus*; [48]), Barramundi (*Lates calcarifer*; [49]), and Brown Trout (*Salmo trutta*; [50]). Also, in our previous fecal microbiota study of *Sacalia*, we found that wild compared to captive individuals had a significantly higher proportion of *Citrobacter*. We suggest that the significant difference of *Citrobacter* between locations is due to diet difference, as Zhang et al. [48] found increases with high-fat diets. Another possibility is an increase in *Citrobacter* due to an increased fruit diet, as proposed by our other study [6]. This pattern seems to hold in this study. Site X has lower *Citrobacter* abundance corresponding to lower fruit abundance; while Sites Y/Z have higher *Citrobacter* abundance corresponding to higher fruit abundance. This hypothesis will need to be tested in other gut microbiota studies.

### Host difference in fecal microbiota

Animals living in the same geographic location tend to have similar gut composition, since geography (abiotic and biotic factors) and diet have influence [36, 40]. By combining our *Platysternon* dataset with a previous study [6], we have a unique opportunity to evaluate the effect of host species (*P. megacephalum* and *S. bealei*) on the fecal microbiota by comparing two syntopic host species. We found that these two species that were captured from the same site at the same time had significantly different fecal microbiotas. At the genus level, the dominant, unique genera were Clostridiales and *Macellibacteroides* in *Platysternon* (Fig. 4D); and *Helicobacter*, *Rhizobacter* and *Comamonas* in *Sacalia* (Fig. 4E). For genera unique to *Platysternon*, the function of Clostridiales is uncertain, while *Macellibacteroides* (as stated previously above) can decompose cellulose- and hemicellulose-derived sugars [43]. For genera unique to *Sacalia*, there is limited information on *Rhizobacter* and *Comamonas*. *Helicobacter* can be commensal in the digestive tract or cause disease in particular hosts [51], and has been inferred to be more host specific, with different *Helicobacter* taxa associated with different vertebrate groups (lizards, turtles, mammals, birds) [52],

Since the environmental microbiota is the same for these two species at Site X, we suggest that the observed fecal microbiota differences are driven by diet preferences and/or host-adapted microbes. These two turtle species have different diet preferences, with *S. bealei* (based on visual fecal analysis and stable isotopes) eat fruit/seeds and terrestrial insects [53], while *P. megacephalum* includes more aquatic resources such as crabs and mollusks [38]. For host-adapted microbes, there may be additional influences from the host (gut physiology, immune system) and microbiota (niche construction, priority effects) [54], but we are unable to differentiate them in this study.

### Functional analysis

As we are sampling microbiota, we expect most of the predicted functions to be related to metabolism, which is the case for all sites and both species (Figure S8). An interesting pattern we found was that the relative abundance of the predicted pathways (level 3 KEGG) was influenced by site and not species; *Platysternon* from Site X were statistically different from *Platysternon* from Sites Y/Z, while both *Platysternon* and *Sacalia* from Site X were statistically indistinguishable. We suggest this pattern be tested in other studies with more species and more localities.

The two most prevalent pathways found in this study were “ABC transporters” and “Two-component system”. Both are categorized under “Environmental Information Processing” in the KEGG level 1 pathways. ABC transporters, also known as ATP-binding cassette transporters, are one of the largest known protein families and widespread throughout all living organisms. These membrane-bound proteins link ATP hydrolysis to active transport, so that a diversity of substrates can be moved in and out of cells. Two-component system is present in bacteria (rare in archaea and eukaryotes), and allows them to respond and adapt to environmental or intracellular change, often by changes in transcription. The majority of the other top 20 pathways (level 3 KEGG) are related to metabolism. This general pattern was also found in other turtle gut microbiota studies [41, 55].

### Turtle gut microbiota and conservation

Studies of turtle gut microbiota are increasing, but relatively few compared to mammals and model organisms [3]. As with other taxa, the early turtle studies have focused on characterizing the gut microbiota of individual species, and the influence of singular factors, such as diet [56, 57], captivity [6, 58], age [57, 59–61], chemicals/pollution [62, 63], and habitat/geography [55, 64–66]. Our study highlights the influence of diet on the intra- and interspecific differences in turtle gut microbiota.

Turtle research is usually connected to conservation because turtles are among the most endangered group of organisms, with over 60% of all species in threatened categories of the IUCN Red List [67]. We believe that gut microbiota research will be an important tool for turtle conservation. It is still unclear what it means to have a healthy microbiota, but in general, higher diversity is related to a healthier host, due to more functional redundancy [68]. Additionally, the microbiota likely has adaptive potential, providing the host functional flexibility [69]. We encourage more researchers to collect gut microbiota data of wild turtles, so we can better understand and apply microbiota data to conservation. For example, care needs to be taken when considering the release of captive and trade turtles, as microbiotas can differ between site (this study) and for individuals in captivity [6, 58]. If there is a mismatch between gut microbiota and geography, released individuals may have lower fitness compared to wild animals [70].

### Supplementary Information

**Additional file 1:** **Figure S2. **Rarefaction curves of samples for *Platysternon*. **Figure S2.** The alpha diversity analysis of *Platysternon* at three sites. **Figure S3.** Comparison of bacterial community at the phylum level. (A) Bar plots of community abundance. Taxa with abundances < 2% have been combined under “others”. (B) Comparison between sites of phyla with abundances > 2%. The phyla significantly different between sites are indicated (**, *P* < 0.01). *P* values are based on Kruskal–Wallis H test or one-way ANOVA, depending on the distribution of the data. **Figure S4.** Comparison of bacterial community at the family level. (A) Bar plots of community abundance. Taxa with abundances < 2% have been combined under “others”. (B) Comparison between sites of the top 5 families based on abundance. The families significantly different between sites are indicated (*, *P* < 0.05; **,* P* < 0.01). *P* values are based on Kruskal–Wallis H test or one-way ANOVA, depending on the distribution of the data. **Figure S5.** Comparison of bacterial community at the genus level. (A) The shared genera across the three sites. (B) Bar plots of community abundance. Taxa with abundances < 2% have been combined under “others”. (C) Comparison between sites of the top 5 genera based on abundance. Genera significantly different between sites are indicated (*, *P* < 0.05).* P* values are based on Kruskal–Wallis H test or one-way ANOVA, depending on the distribution of the data. **Figure S6.** Linear discriminant analysis effect size (LEfse) (A), The bar graph of LDA scores showing the taxa statistically different between Sites X, Y, and Z. The degree of influence of a taxon is expressed by the length of the bar. Only taxa meeting an LDA significant threshold > 2 are shown. (B) Cladogram of taxa showing significant difference between sites. Red, blue, and green dots represent the core bacterial populations in Sites X, Y, and Z, respectively. **Figure S7**. The alpha diversity analysis of *Platysternon* and *Sacalia* at Site X. **Figure S8.** The relative abundance of different functional pathways of the gut microbiota. The heat map shows the predicted pathways of KEGG level 1. The value shows the percentage of pathway abundance.

## Data Availability

The 16S rRNA sequence data about *P. megacephalum* and *S. bealei* reported in this study have been deposited in NCBI Sequence Read Archive (SRA) database, under accession number PRJNA824218 and PRJNA623155, respectively.
