# Supplementary material for: Fine-scale geographic difference of the endangered Big-headed Turtle (Platysternon megacephalum) fecal microbiota, and comparison with the syntopic Beale’s Eyed Turtle (Sacalia bealei)
Source: BMC Microbiol. 2024 Feb 29;24:71. doi: 10.1186/s12866-024-03227-2 (PMC10902975; doi:10.1186/s12866-024-03227-2)
Supplement: Supplementary file 1 — Additional file 1: Figure S2. Rarefaction curves of samples for Platysternon. Figure S2. The alpha diversity analysis of Platysternon at three sites. Figure S3. Comparison of bacterial community at the phylum level. (A) Bar plots of community abundance. Taxa with abundances < 2% have been combined under “others”. (B) Comparison between sites of phyla with abundances > 2%. The phyla significantly different between sites are indicated (**, P < 0.01). P values are based on Kruskal–Wallis H test or one-way ANOVA, depending on the distribution of the data. Figure S4. Comparison of bacterial community at the family level. (A) Bar plots of community abundance. Taxa with abundances < 2% have been combined under “others”. (B) Comparison between sites of the top 5 families based on abundance. The families significantly different between sites are indicated (*, P < 0.05; **, P < 0.01). P values are based on Kruskal–Wallis H test or one-way ANOVA, depending on the distribution of the data. Figure S5. Comparison of bacterial community at the genus level. (A) The shared genera across the three sites. (B) Bar plots of community abundance. Taxa with abundances < 2% have been combined under “others”. (C) Comparison between sites of the top 5 genera based on abundance. Genera significantly different between sites are indicated (*, P < 0.05). P values are based on Kruskal–Wallis H test or one-way ANOVA, depending on the distribution of the data. Figure S6. Linear discriminant analysis effect size (LEfse) (A), The bar graph of LDA scores showing the taxa statistically different between Sites X, Y, and Z. The degree of influence of a taxon is expressed by the length of the bar. Only taxa meeting an LDA significant threshold > 2 are shown. (B) Cladogram of taxa showing significant difference between sites. Red, blue, and green dots represent the core bacterial populations in Sites X, Y, and Z, respectively. Figure S7. The alpha diversity analysis of Platysternon and Sacalia at Site X. Fi [file 12866_2024_3227_MOESM1_ESM.docx]

**Table S1** General information for each sample. The first letter in the name (X, Y, Z) indicates the group based on locality of *Platysteronon* samples, while other samples (WS) represent *Sacalia* samples.

| **Sample** | **Age class & sex** | **Carapace length (mm)** | **Sequencing** | | | | |
| --- | --- | --- | --- | --- | --- | --- | --- |
|  |  |  | **Number of reads** | **Number of bases** | **Mean length** | **Min length** | **Max length** |
| X1 | Juvenile | 84.0 | 46,409 | 19,104,222 | 412 | 262 | 474 |
| X2 | Adult female | 119.4 | 51,251 | 21,105,758 | 412 | 345 | 442 |
| X3 | Adult female | 112.5 | 59,211 | 24,542,940 | 415 | 262 | 430 |
| X4 | Adult female | 107.5 | 46,742 | 19,588,545 | 419 | 262 | 446 |
| Y1 | Adult male | 120.1 | 51,338 | 21,357,751 | 416 | 214 | 431 |
| Y2 | Juvenile | 93.3 | 50,950 | 21,328,809 | 419 | 307 | 431 |
| Y3 | Juvenile | 68.2 | 45,378 | 19,113,939 | 421 | 262 | 431 |
| Y4 | Juvenile | 68.5 | 39,710 | 16,539,131 | 417 | 361 | 431 |
| Y5 | Adult male | 131.3 | 40,588 | 17,368,559 | 428 | 345 | 432 |
| Y6 | Adult male | 114.3 | 50,529 | 21,178,286 | 419 | 252 | 431 |
| Y7 | Juvenile | 74.2 | 38,910 | 16,527,723 | 425 | 402 | 431 |
| Z1 | Adult male | 153 | 43,047 | 18,033,798 | 419 | 261 | 461 |
| Z2 | Adult male | 103.7 | 52,494 | 21,395,792 | 408 | 209 | 431 |
| Z3 | Juvenile | 59.2 | 37,359 | 15,797,241 | 423 | 401 | 430 |
| Z4 | Adult female | 118.5 | 40,908 | 17,053,758 | 417 | 262 | 430 |
| Z5 | Adult male | 120.1 | 54,394 | 22,885,245 | 421 | 337 | 431 |
| WS1.1 | Juvenile | 58.5 | 62,393 | 25,821,244 | 414 | 261 | 431 |
| WS1.2 | Adult female | 111.0 | 54,102 | 22,669,203 | 419 | 400 | 433 |
| WS1.3 | Adult female | 120.1 | 50,552 | 211,00,822 | 417 | 249 | 432 |

**Figure S1** Rarefaction curves of samples for *Platysternon*.

**Figure S2** The alpha diversity analysis of *Platysternon* at three sites.

**Figure S3 Comparison of bacterial community at the phylum level.** (A) Bar plots of community abundance. Taxa with abundances < 2% have been combined under “others”. (B) Comparison between sites of phyla with abundances > 2%. The phyla significantly different between sites are indicated (**, *P* < 0.01). *P* values are based on Kruskal-Wallis H test or one-way ANOVA, depending on the distribution of the data.

**Figure S4 Comparison of bacterial community at the family level.** (A) Bar plots of community abundance. Taxa with abundances < 2% have been combined under “others”. (B) Comparison between sites of the top 5 families based on abundance. The families significantly different between sites are indicated (*, *P* < 0.05; **, *P* < 0.01). *P* values are based on Kruskal-Wallis H test or one-way ANOVA, depending on the distribution of the data.

**
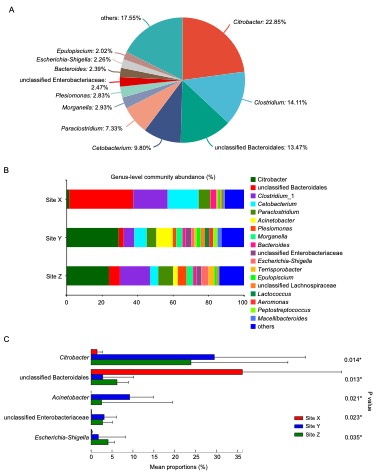
**

**Figure S5 Comparison of bacterial community at the genus level.** (A) The shared genera across the three sites. (B) Bar plots of community abundance. Taxa with abundances < 2% have been combined under “others”. (C) Comparison between sites of the top 5 genera based on abundance. Genera significantly different between sites are indicated (*, *P* < 0.05). *P* values are based on Kruskal-Wallis H test or one-way ANOVA, depending on the distribution of the data.

**
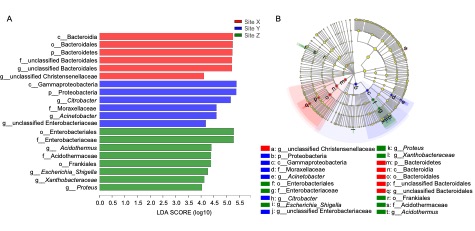
**

**Figure S6 Linear discriminant analysis effect size (LEfse)** (A), The bar graph of LDA scores showing the taxa statistically different between Sites X, Y, and Z. The degree of influence of a taxon is expressed by the length of the bar. Only taxa meeting an LDA significant threshold > 2 are shown. (B) Cladogram of taxa showing significant difference between sites. Red, blue, and green dots represent the core bacterial populations in Sites X, Y, and Z, respectively.

**Figure S7** **The alpha diversity analysis of *Platysternon* and *Sacalia* at Site X**

**Figure S8 The relative abundance of different functional pathways of the gut microbiota.** The heat map shows the predicted pathways of KEGG level 1. The value shows the percentage of pathway abundance.
